# Prognostic Significance of SPARC Expression in Breast Cancer: A Meta-Analysis and Bioinformatics Analysis

**DOI:** 10.1155/2022/8600419

**Published:** 2022-02-15

**Authors:** Shuai Shi, Hong-Yan Ma, Xin-Ying Han, Yin-Zhou Sang, Ming-Yue Yang, Zhi-Gang Zhang

**Affiliations:** Department of Pathology, Cangzhou People's Hospital, Cangzhou 061000, China

## Abstract

Secreted protein, acidic and rich in cysteine (SPARC, also known as osteonectin), is a small molecule glycoprotein associated with cell secretions. The purpose of our research is to clarify the clinicopathological and prognostic significance of SPARC expression in breast cancer. In this study, we performed a meta-analysis and bioinformatics analysis using the PubMed, Web of Science, Wanfang Data, and CNKI databases. The meta-analysis showed that SPARC expression was elevated in breast cancer tissue, compared with normal tissue, while SPARC expression in tumor stromal cells was higher than that of tumor cells. The expression of SPARC was positively correlated with histological grade and TNM staging. The Kaplan-Meier plotter showed that low SPARC expression was negatively correlated with the overall, postprogression, and distant metastasis survival rates of patients. According to Oncomine database, SPARC expression was upregulated in breast cancer than normal tissues. In TCGA database, univariate analysis showed that lymph node metastasis, distant metastasis, and TNM staging were negatively correlated with patient prognosis in breast cancers. Cox multivariate analysis showed that age, lymph node metastasis, distant metastasis, and TNM staging were important factors affecting the survival time of breast cancer patients. SPARC expression can be employed as a good indicator of prognosis of breast cancer patients, which will provide new methods and ideas of preventive treatment.

## 1. Introduction

Secreted protein, acidic and rich in cysteine (SPARC, also known as osteonectin), is a small molecule glycoprotein associated with cell secretions [1]. The SPARC gene is located on human chromosome Sq31.3-q32 and contains 10 exons, while the protein contains 298-304 amino acids. SPARC contains three highly conserved domains: the amino-terminal acidic calcium ion domain, the copper ion region homologous to the follicular dormancy hormone, and the extracellular calcium ion region [2, 3]. Osteopontin (SPARC) is involved in embryonic development, tissue repair, and cell regeneration. SPARC can regulate cell adhesion and cell proliferation through different signaling pathways and is a highly conserved extracellular interstitial protein. Its main functions are to prevent cell adhesion, regulate cell differentiation, prevent the spreading of cells, inhibit cell response to specific growth factors, regulate extracellular matrix and matrix metalloproteinase production, and influence neovascularization [4, 5]. Many of the biological functions of SPARC are stimulated by interactions with other proteins. Interactions with different proteins can produce a range of functional activities leading to diverse SPARC functions [6]. For example, SPARC participates in the regulation of the extracellular matrix. Its interaction with collagen affects the remodeling of connective tissue.

Additionally, glycoprotein and hyaluronectin contain the same expression site as SPARC, but the two proteins produce opposite effects on cell adhesion and are also associated with tissue remodeling [7]. SPARC can directly bind to vascular endothelial growth factor (VEGF) to inhibit the vascular endothelial growth factor pathway, preventing vascular endothelial growth factor and its receptor from binding to each other [8]. At the same time, SPARC can also bind to platelet-derived growth factor PDGF to indirectly inhibit angiogenesis by downregulating matrix metalloproteinases (MMPs) and transforming growth factor *β*1 antibodies (TGF-*β*1), which in turn inhibits tumor invasion and metastasis [9, 10]. Abnormal methylation of the CpG island of the promoter region of the SPARC gene can inhibit SPARC expression [11].

Abnormal methylation of the SPARC gene promoter region that leads to gene expression silencing has been observed in primary pancreatic cancer. SPARC mRNA is expressed in nonneoplastic pancreatic ductal epithelial cells but is not found in pancreatic cancer cell lines, which indirectly indicates that the silencing of the SPARC gene can lead to the development of pancreatic cancer [12, 13]. These findings suggest that the abnormal methylation of the CpG 2 region may be a suitable marker that can be used for the preliminary screening of early pancreatic cancer. SPARC increases the level of phosphorylation of AKT in gliomas through the PI3K/AKT pathway, significantly inhibiting EGF activity in ovarian cancer [14], and can activate GSK3*β* targets in adipocytes [15]. In mesangial cells, SPARC can enhance the activity of TGF-*β*, which leads to an increase in JNK activation and c-Jun phosphorylation [16]. In the Wnt pathway, SPARC can activate ILK, thereby phosphorylating its downstream in GSK-3B target. Phosphorylation of this target can lead to *β*-catenin aggregation and ultimately TCF4 activation. This pathway can also inhibit the expression of laminin in melanocytes [17, 18].

Previous research has shown that SPARC is closely associated with tumor development and plays a vital role in tumor invasion and metastasis [19]. SPARC expression is elevated in melanoma, glioma, meningioma, kidney cancer, and prostate cancer [20–23]. High level of SPARC expression enhanced tumor invasion and metastasis, leading to a poor prognosis of patients. In our study, we performed a meta-analysis and bioinformatics analysis to confirm the relationship between SPARC mRNA expression and the clinicopathological factors of breast cancer.

## 2. Materials and Methods

### 2.1. Static Search and Data Extraction

Articles were searched in PubMed, Web of Science, Wanfang Data, and CNKI databases (May 2020) using the key words: SPARC and breast and cancer or carcinoma or tumor. Inclusion criteria for studies included (1) breast cancer patients; (2) expression of SPARC was detected by immunohistochemistry; (3) articles contain SPARC expression and clinicopathological parameters; (4) all patients did not receive chemotherapy or radiotherapy before surgery. Exclusion criteria included (1) abstracts, case reports, reviews, and meeting notes; (2) repeat publications; (3) unclear diagnosis.

### 2.2. Data Extraction and Quality Assessment

As shown in Table 1, the information of eligible publications was extracted by two reviewers (Shi S and Zhang ZG) and included name of the first author, year of publication, patients' country, antibody company, number of cases and controls, risks for cancer, and follow-up outcomes. According to the Newcastle Ottawa Oncomine Scale (NOS; ohri.ca/programs/clinical_epidemiology/oxford.htm), the quality of the studies was independently assessed by two reviewers. The methods consists sample selection, comparability, and ascertainment of outcomes.

### 2.3. Bioinformatics Analysis

The prognostic value of SPARC mRNA expression in breast cancer was analyzed using a Kaplan-Meier plotter (http://www.kmplot.com). Expression of SPARC was associated with overall survival (OS), relapse-free survival (RFS), distant metastasis-free survival (DMFS), and postprogression survival (PPS) for all patients. The expression of SPARC was associated with clinicopathological features. SPARC gene expression was analyzed using the Oncomine database (http://www.oncomine.org), an extensive database of tumor chip data, including gene chip and gene expression data. The database can be used to analyze gene expression differences and classify the clinical information of tumor patients. Differences in SPARC expression at mRNA level were compared between carcinoma and normal tissues. Data on gene expression and the clinical pathology of SPARC were downloaded from the cancer genome atlas (TCGA, http://www.cancer.gov) database using the TCGA-assembler of R software. The letter code of breast cancer is BRCA. We organized the data and analyzed the mRNA expression of SPARC in breast cancer. At the same time, we analyzed the clinicopathological data and prognosis of tumor patients. Cox risk regression models were used to conduct univariate and multivariate analyses. This model analyzed the effect of risk factors, the hazard ratio (HR), and 95% confidence interval (CI).

### 2.4. Statistical Analysis

Revman version 5.3 was used for the data analysis. The results of the comparison between the case group and the control group were expressed as an odds ratio (OR) and 95% CI. *I*^2^ statistics were used to determine the heterogeneity between the research results. If a significant level of heterogeneity was found, a fixed-effect model (*I*^2^ < 50%, *P* > 0.10, Mantel-Haenszel method) was used; otherwise, a random effect model (*I*^2^ ≥ 50%, *P* ≤ 0.10, Der Simonian and Laird method) was used. Publication bias was evaluated using a funnel plot, and funnel plot asymmetry was quantified using Begg's test and Egger's test. Cox risk regression models were used for the univariate and multivariate analyses. This model analyzed the effect of risk factors, the hazard ratio, and 95% CI. A *P* value of <0.05 was considered to indicate a statistically significant difference. All data analyses were conducted using SPSS 19.0 software.

## 3. Results

### 3.1. Study Selection and Characteristics

**Figure 1:**
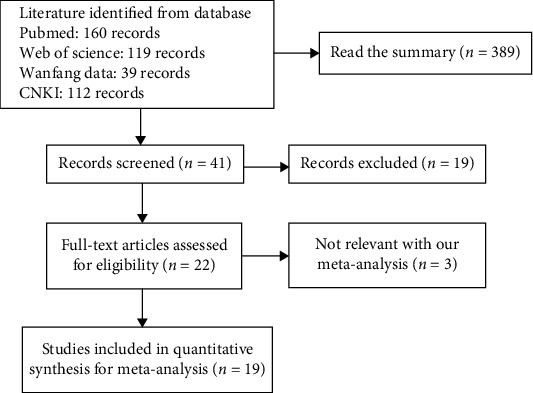
Flow diagram of article selection.

As shown in Figure 1 and Table 1, a total of 20 articles that analyzed the relationship between SPARC expression and the clinicopathological characteristics of breast cancer were identified [24–44]. However, only 10 of these articles contained an analysis of normal breast tissues [24, 28–31, 38–42]. Data on the clinicopathological characteristics of breast cancer included histological grade, TNM staging, lymph node metastasis, menopausal status, tumor size, and the presence of ER, PR, and HER2 (Table 2). Finally, only 4 articles were found to include the prognostic features of SPARC expression and its relationship with breast cancer [25, 27, 29, 37].

### 3.2. Forest Plot of OR for the Association between SPARC Expression and the Clinicopathological Characteristics of Breast Cancer

**Figure 2:**
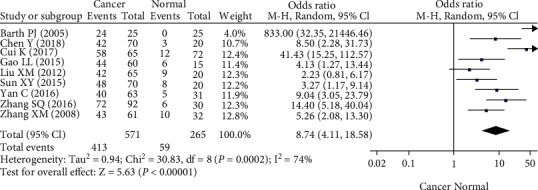
Forest plot of the expression of SPARC in breast cancer. Plots of the association between cancer and normal mucosa.

A total of 10 articles that included data on 571 breast cancer patients and 265 normal controls were found. The expression of SPARC was upregulated in breast cancer tissue compared with normal tissues (Figure 2). Our meta-analysis showed that SPARC expression was associated with TNM staging and histological grade (Table 2). The expression of SPARC was also upregulated in breast stromal cells, compared with tumor cells (Table 2).

### 3.3. Publication Bias

**Figure 3:**
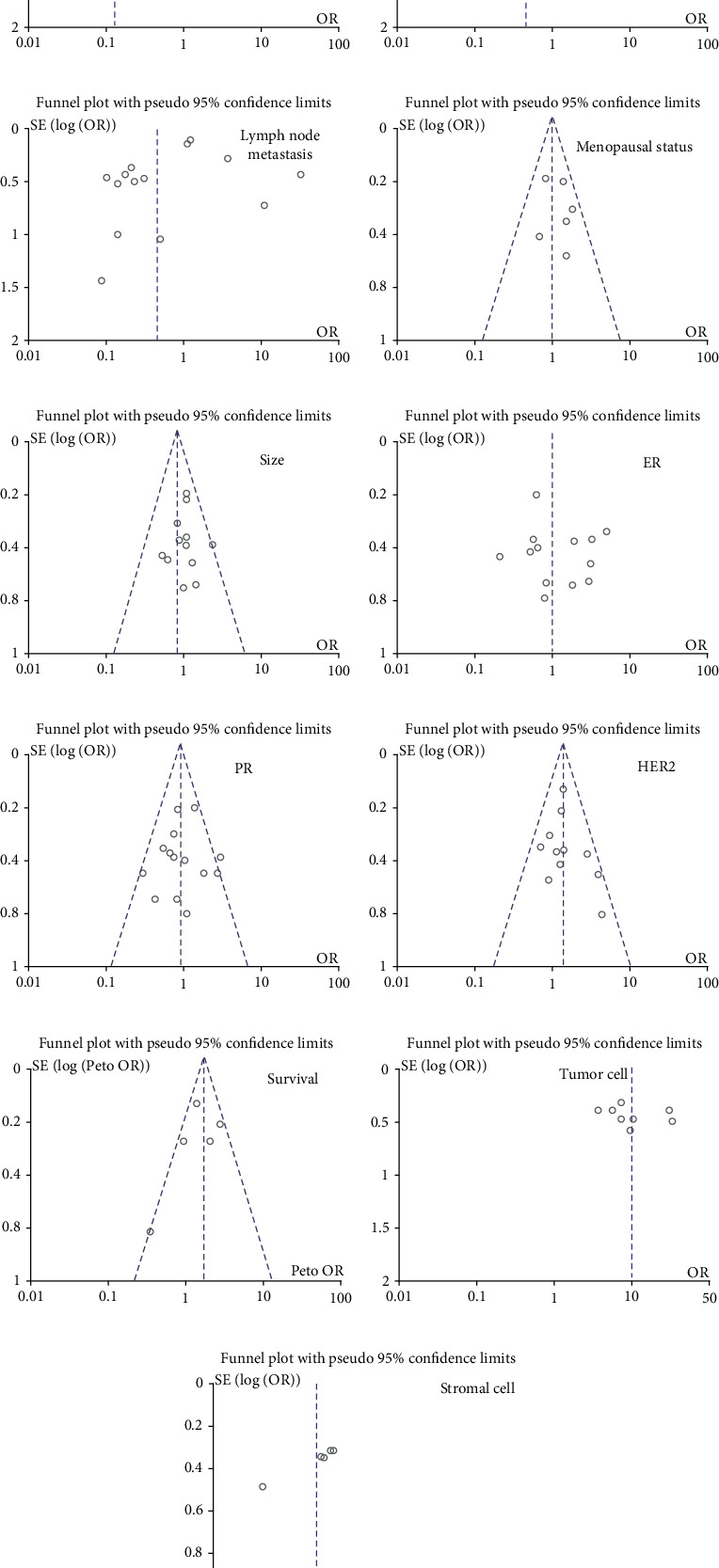
Funnel plot for testing publication bias between SPARC expression and breast cancer. Publication bias was also tested between SPARC expression and clinicopathological features of breast cancer, including (a) histological grade, (b) TNM staging, (c) lymph node metastasis, (d) Menopausal status, (e) size, (f) ER, (g) PR, (h) HER2, and (i) survival. Additionally, publication bias was analyzed based on risk degrees of SPARC expression in (a) tumor cell and (b) stromal cell.

As shown in Figure 3, the heterogeneity between studies was tested using funnel diagrams. Each study was removed from the pooled analysis to assess the impact of each individual study on the aggregated results using a sensitivity analysis. Egger's test results showed that there was no apparent publication bias in this meta-analysis.

### 3.4. The Relationship between SPARC Expression and the Bioinformatics Features of Breast Cancer

**Figure 4:**
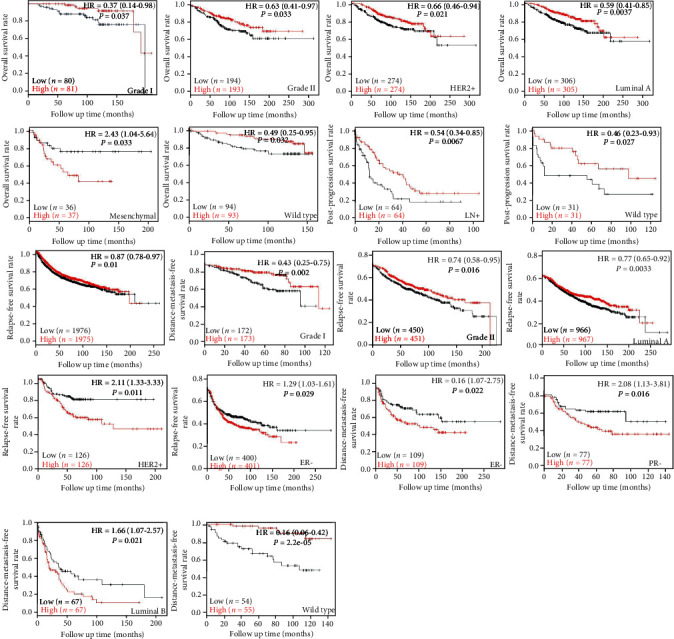
Prognostic value of SPARC mRNA expression in breast cancer patients according to KM-plotter (http://www.kmplot.com).

**Figure 5:**
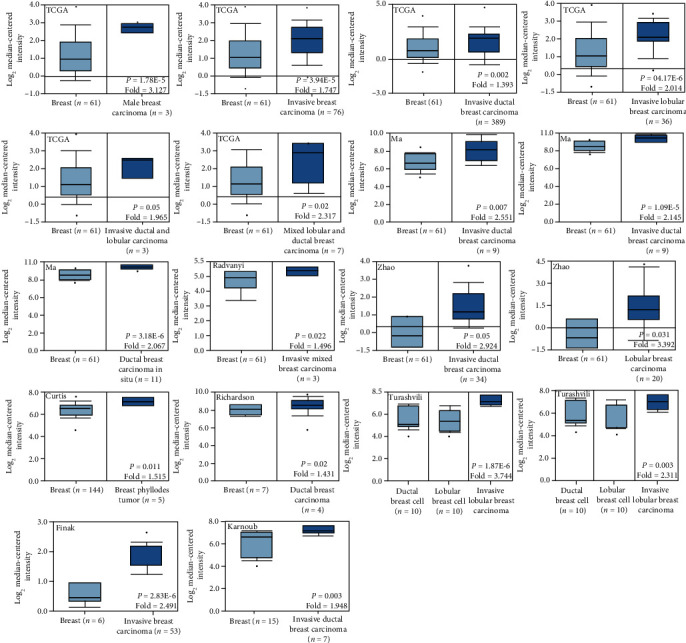
Prognostic value of SPARC mRNA expression in breast cancer patients according to Oncomine database (http://www.oncomine.org).

The Kaplan-Meier plotter was used to find that lower SPARC expression was negatively correlated with the overall survival rate of grade I/II, Her2+, luminal A, wild type patients, the postprogression survival rate of LN+, wild type patients, and the distant metastasis survival rate of wild-type patients (Figure 4, *P* < 0.05). Elevated SPARC expression was positively correlated with the relapse-free survival rate of patients, even when stratified as grade I/II and luminal A patients, but an opposite result was obtained when stratified as Her2+, ER patients. ER, PR, or luminal B patients and elevated SPARC expression produced a shorter distant metastasis survival rate, compared with a lower expression level (Figure 4, *P* < 0.05). Based on the TCGA analysis, as well as the databases published by Ma, Radvanyi, Zhao, Curtis, Richardson, Turashvil, Final, and Karnoub, we found that SPARC expression was lower in breast tissues than in invasive or ductal breast carcinoma, invasive lobular, ductal or mixed breast carcinoma, and breast phyllodes tumors. Elevated SPARC expression was found in invasive ductal or lobular breast carcinomas, compared with ductal or lobular breast carcinomas (Figure 5, *P* < 0.05). The Cox univariate analysis of TCGA data showed that age, TNM staging, lymph node metastasis, and distant metastasis were negatively correlated with patient prognosis (Table 3, *P* < 0.05). The Cox multivariate analysis showed that age, TNM staging, lymph node metastasis, and distant metastasis found to be risk factors that for breast cancer patient prognosis (Table 4, *P* < 0.05).

## 4. Discussion

SPARC acts as an antiadhesion protein to regulate growth factors and matrix proteases. SPARC can counteract the effect of *β*-FGF in promoting cell proliferation, migration, and mediating angiogenesis, while it can also inhibit *β*-FGF receptor autophosphorylation and ERK 1/2 activation [43]. Additionally, SPARC can inhibit VEGF-mediated endothelial cell proliferation and directly bind to PDGF-B and interfere with its binding to fibroblast receptors [44, 45]. TGF-*β* can mediate the production of SPARC proteins. With low levels of TGF-*β*, expression levels have been found in the glomerular mesangial cells of SPARC knockout mice [3]. SPARC can mediate the expression of MMP-1 and MMP-9 in peripheral blood monocytes and the expression of MMP-1, MMP-3, and MMP-9 in fibroblasts [46].

Studies have found that SPARC is mainly expressed in mesenchymal cells in gastric cancer tissue. The expression of SPARC was negatively correlated with differentiation, Lauren classification, lymph node metastasis, and clinical stage, while lymph node metastasis was an independent prognostic factor for patients with gastric cancer [47–49]. Sato et al. found that SPARC expression at mRNA level in cancer tissues was significantly higher than that of normal adjacent tissues [50]. Previous research has shown that SPARC methylation occurs at a high rate in gastric cancer tissues and that promoter DNA methylation can inhibit SPARC expression in gastric cancer cells [51]. SPARC was also highly expressed in esophageal and liver cancer tissues and was closely related to the degree of malignancy, but its expression was low in pancreatic and colon cancer tissues [13, 15, 52, 53]. SPARC expression was significantly lower in ovarian cancer tissues than in normal tissues and in patients with a poorly differentiated and larger omentum [54]. SPARC expression could inhibit the production of interleukin-6 in ovarian cancer tissues and decrease levels of peritoneal metastasis caused by ovarian cancer. SPARC could also block the transformation of vascular endothelial cells from the G1 phase to the S phase and induce the apoptosis and migration of vascular endothelial cells [55, 56]. Similar results were observed in endometrial cancer but not in cervical cancer [57, 58]. SPARC expression was also higher in lung squamous cell carcinoma than in lung adenocarcinoma. SPARC was found to be synthesized and secreted by tumor stromal cells [59], and its expression was associated with an acidic environment and the necrosis of tumor tissues, as its expression levels were higher in tumor necrosis areas [60]. The results of multivariate and univariate analyses demonstrated the prognostic value of SPARC expression in determining the overall survival rate [59].

SPARC protein expression was significantly associated with interstitial remodeling, the loss of CD34, and *α*-SMA expression in invasive ductal carcinoma and interfered with TGF-*β*1 signaling, which allowed it to play a role in tumor progression [38]. SPARC was highly expressed in breast cancer tissues and was associated with TNM staging and lymph node metastasis. The Cox analysis showed that TNM staging and lymph node metastasis were risk factors that affected the prognosis of patients. RT-PCR results showed that SPARC was highly expressed at the mRNA level in breast cancer tissues [61, 62]. Our research results also elevated that high SPARC expression was associated with TNM staging and histological grade. The univariate analysis showed that age, TNM staging, lymph node metastasis, and distant metastasis were associated with a poor prognosis in breast cancer patients. The analysis conducted using the Cox proportional hazard regression model showed that age, TNM staging, lymph node metastasis, and distant metastasis are essential factors that affect the survival time of breast cancer patients.

SPARC expression at protein and mRNA levels showed opposite trends in breast cancer. The mRNA level expression of a particular gene does not necessarily have a linear relationship with the expression level of its translated product protein due to several reasons. First, gene expression is regulated at various levels, and regulation at the transcription level is only an intermediate link. Second, posttranscriptional translation and posttranslational regulation all contribute to the expression of the final protein. Finally, the mRNA degradation, protein degradation, and modified folding may cause inconsistencies between mRNA and protein expression levels of a given protein.

## 5. Conclusion

SPARC plays a complex role in tumorigenesis and development. At the same time, SPARC expression is upregulated in breast cancer patients. SPARC is positively related with TNM staging and histological grade of breast cancer patients. SPARC expression can be employed as a good marker for the prognosis of patients with cancers, which will provide new methods and ideas for preventive treatment.

## Figures and Tables

**Table 1:** Main characteristics of eligible studies.

First author	Year	Country	Ethnicity	Antibody supplier	Cases	Ctr	Risk to cancer	Outcome	Quality	Reference
Cui K	2017	China			65	72	Increased		8	24
Xu XD	2014	China	UK	Abcam	255			Negative	7	25
Zhou QF	2020	China	UK	Abcam	150				7	26
Chen F	2017	China	USA	Cell Signaling	122			Negative	7	27
Yan C	2016	China			63	31	Increased		8	28
Chen Y	2018	China	China	Bioss	70	20	Increased	Negative	8	29
Gao LL	2015	China	China	Bioss	60	15	Increased		8	30
Zhang XM	2008	China	USA	Santa	61	32	Increased		8	31
Lindner JL	2014	German	UK	Novocastra	667				8	32
Ma JJ	2017	China	USA	Cell Signaling					7	33
Zhu AJ	2016	China	USA	Thermo	211				7	34
Guo W	2017	China	UK	Abcam	88				8	35
Witkiewicz AK	2010	USA	Denmark	Dako					7	36
Watkins G	2005	UK	USA	Santa				Negative	7	37
Barth PJ	2005	German	UK	Novocastra	25	25	Increased		8	38
Sun XY	2015	China	China	Bioss	70	20	Increased		8	39
Liu XM	2012	China	China	Bioss	65	20	Increased		8	40
Zhang SQ	2016	China	China	Bioss	92	30	Increased	Negative	8	41
Sun XY	2014	China	China	Bioss	70	20	Increased		8	42

**Table 2:** Results of meta-analysis of the correlation between SPARC expression and clinical pathological features of breast cancer.

Clinicopathological features	Heterogeneity		Test for overall effect	
	*I* ^2^ (%)	*P* value	Odds ratio (95% CI)	*P* value
Histological grade	45	0.08	0.66 (0.43-1.00)	0.05^∗^
TNM staging	61	<0.01	0.47 (0.31-0.71)	<0.01^∗^
Lymph node metastasis	85	<0.01	0.52 (0.25-1.05)	0.07
Menopausal status	0	0.49	1.03 (0.76-1.40)	0.85
Size	0	0.92	0.86 (0.67-1.12)	0.27
ER	67	<0.01	0.98 (0.59-1.63)	0.94
PR	27	0.17	0.78 (0.60-1.02)	0.07
HER2	0	0.45	1.08 (0.84-1.37)	0.56
Overall survival	63	0.05	1.27 (0.86-1.89)	0.23
SPARC tumor cell	74	<0.01	8.74 (4.11-18.58)	<0.01^∗^
SPARC stromal cell	65	0.02	0.18 (0.10-0.33)	<0.01^∗^

TNM: tumor node metastasis; ER: estrogen receptor; PR: progesterone receptor; HER2: human epidermal growth factor receptor-2.

**Table 3:** Univariate analysis of prognostic risk factors in the patients with breast cancer.

Characteristics	Patients (%)	Relative risk (95% CI)	*P* value
Sex			
Female	1065 (98.9)	0.852 (0.119-6.102)	0.873
Male	12 (1.1)		
Age(years)			
<60	599 (56.0)	0.516 (0.371-0.719)	<0.001^∗^
≥60	470 (44.0)		
TNM staging			
I-II	792 (73.4)	0.384 (0.272-0.543)	<0.001^∗^
III-IV	259 (24.6)		
Depth of invasion			
-	281 (26.2)	0.734 (0.497-1.084)	0.120
+	792 (73.8)		
Lymph node metastasis			
-	504 (47.6)	0.468 (0.352-0.672)	<0.001^∗^
+	555 (52.4)		
Distant metastasis			
-	893 (97.6)	0.208 (0.124-0.349)	<0.001^∗^
+	22 (2.4)		

CI: confidence interval; TNM: tumor node metastasis.

**Table 4:** Multivariate analysis of clinicopathological variables for the survival of the patients with breast cancer.

Clinicopathological parameters	Relative risk (95% CI)	*P*
SPARC expression (+)	0.855 (0.597-1.223)	0.390
Age (≥60 years)	2.070 (1.425-3.007)	<0.001^∗^
Sex (female)	1.753 (0.242-12.705)	0.578
Depth of invasion (T2-4)	1.123 (0.709-1.778)	0.622
Lymph node metastasis (+)	1.622 (1.026-2.563)	0.038^∗^
Distant metastasis (+)	2.547 (1.310-4.950)	0.006^∗^
TNM staging (III–IV)	1.707 (1.063-2.742)	0.027^∗^

CI: confidence interval; TNM: tumor node metastasis.
